# The identification of a novel splicing mutation in *C1qB* in a Japanese family with C1q deficiency: a case report

**DOI:** 10.1186/1546-0096-11-41

**Published:** 2013-10-28

**Authors:** Yousuke Higuchi, Junya Shimizu, Michiyo Hatanaka, Etsuko Kitano, Hajime Kitamura, Hidetoshi Takada, Masataka Ishimura, Toshiro Hara, Osamu Ohara, Kenji Asagoe, Toshihide Kubo

**Affiliations:** 1 Department of Pediatrics, National Hospital Organization Okayama Medical Center, 1711-1 Tamasu, Kita-ku, Okayama 701-1192, Japan; 2 Department of Medical Technology Faculty of Health Sciences, Kobe Tokiwa University, 2-6-2 Ohtanicho, Nagata-ku, Kobe 653-0838, Japan; 3 Department of Pediatrics, Graduate School of Medical Sciences, Kyushu University, 3-1-1 Maidashi, Higasi-ku, Fukuoka 812-8582, Japan; 4 Department of Human Genome Technology, Kazusa DNA Research Institute, 2-6-7 Kazusakamatari, Chiba 292-0818, Japan; 5 Department of Dermatology, National Hospital Organization Okayama Medical Center, 1711-1 Tamasu, Kita-ku, Okayama 701-1192, Japan

**Keywords:** C1q deficiency, Systemic lupus erythematosus, Hypocomplementemia, Novel mutation, Fresh frozen plasma

## Abstract

C1q deficiency is a rare disease that is associated with a high probability of developing systemic lupus erythematosus. We report a 4-year-old Japanese girl who presented with fever, facial erythema, joint pain, and oral ulceration. Complement deficiencies were suspected because of her persistent hypocomplementemia and normal levels of the complement proteins C3 and C4. We identified a novel homozygous splicing mutation in the *C1qB* gene, c.187 + 1G > T, which is the first mutation to be confirmed in a Japanese individual. Because treatment with steroids and immunosuppressive drugs was not effective, we commenced use of fresh frozen plasma to provide C1q supplements. Currently, the patient remains almost asymptomatic, and we are attempting to control the drug dosage and administration intervals of fresh frozen plasma.

## Background

The complement system involves a group of proteins that function as part of the immune system. Three complement pathways make up the complement system, the classical, alternative, and lectin pathways. The C1q protein is the first component of the classical pathway and is composed of the C1qA chain, C1qB chain, and C1qC chain, which are encoded by *C1qA*, *C1qB*, and *C1qC* genes, respectively [1].

C1q deficiency is a rare disease that is associated with a high probability of developing systemic lupus erythematosus (SLE) [2,3]. It is also complicated by cutaneous disease, glomerulonephritis, central nerve system lupus, and recurrent bacterial infection at an early age [2,4]. A deficiency of other complement components such as C1r, C1s, C2, and C4 is also involved in SLE, with C1q deficiency being the strongest single risk factor for SLE development [5]. The first C1q deficiency-causing mutation was reported by McAdam et al. in 1988 [6]. Today, 14 mutations have been identified, all of which are nonsense or missense mutations [7-9]. Although immunosuppressive therapy is administered to C1q deficiency patients, there is currently no curative therapy.

We report herein a girl with C1q deficiency, and the identification of a novel splice site mutation in the *C1qB* gene, which is the first confirmed genetic mutation in a Japanese individual with C1q deficiency.

## Case presentation

A 4-year-old Japanese girl was referred to our hospital with a three month history of fever, facial erythema, joint pain, and oral ulceration. She had been diagnosed with discoid lupus erythematosus following a skin biopsy at another hospital. Her symptoms were alleviated transiently after the oral administration of prednisolone; however, they relapsed after cessation of treatment. She had no past history of recurrent infections or fevers of unknown origin. Her parents are not consanguineous, and all other family members, including parents and two brothers, were healthy and had not complained of SLE-like symptoms at that time.

**Figure 1:**
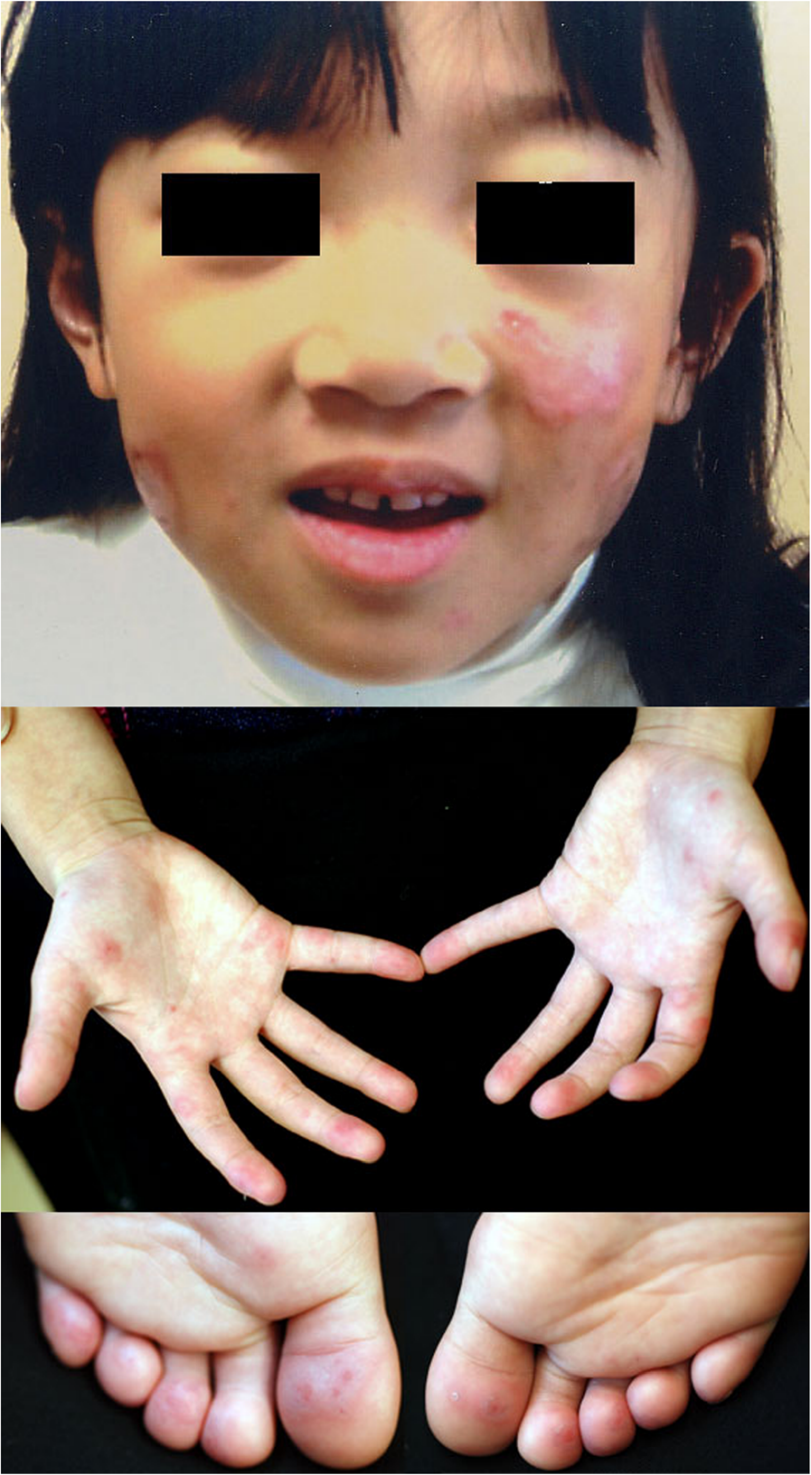
Lupus erythematosus malar skin rash and rash on the hand and foot of our patient.

At the first visit to our department, physical examinations revealed low grade fever, scarring facial erythema, oral ulceration, and a chilblain-like rash on the extremities of her limbs (Figure 1). Breath sounds were clear and unlabored. Cardiac examination revealed no murmurs, rubs, or gallops. The abdomen was soft, nontender, and nondistended. Neurological examinations of the cranial nerve, motor strength and coordination, reflexes, gait and sensation were normal. Blood and urine tests for rheumatic fever and other febrile illness revealed elevated inflammatory markers (erythrocyte sedimentation rate (ESR) 65 mm/60 min, C-reactive protein (CRP) 1.32 mg/dl), although blood cell count, electrolytes, blood urea nitrogen, creatinine, liver function, and urinary tests were all normal (Table 1). Total complement activity (CH50) was not detectable but an immunoturbidimetric assay revealed that C3 and C4 levels were within the normal range. Speckled antinuclear antibody and rheumatoid factor were positive, and anti-double stranded DNA IgG antibody levels were negative. A chest computed tomography (CT), echocardiography, magnetic resonance imaging of the brain, and renal biopsy specimen were normal.

**Table 1:** Laboratory findings on first visit of patient

**Blood test**	**Result**	**Normal range**	**Blood test**	**Result**	**Normal range**
WBC	6600/μl	4500–15500	CRP	1.32 mg/dl	<0.1
Seg	5600/μl	1500–8500	ESR 60 min	65 mm/60 min	0–15
Ly	660/μl	1200–8000			
RBC	379 × 10^4^/μl	390–490	CH50	<12.0 CH50/ml	22–40
Hb	10.7 g/dl	11.4–14.2	C3	122 mg/dl	71–159
Ht	30.5%	34–40	C4	45 mg/dl	13–30
Plt	15.2 × 10^4^/μl	14.0–45.0	RF	31 IU/ml	<40
AST	31 IU/l	18–63	ANA(speckled)	1:320	<1:40
ALT	21 IU/l	20–50	Anti-dsDNA IGG	<10 IU/ml	<10
LDH	297 IU/l	142–297	Anti-Sm	1:2	<1:2
Na	141 mEq/l	134–143	Anti-RNP	1:16	<1:2
K	3.3 mEq/l	3.4–4.9	Anti-Ro	(-)	(-)
Cl	104 mEq/l	98–107	Anti-La	(-)	(-)
Ca	9.0 mg/dl	8.8–10.3			
TP	6.3 g/dl	5.6–7.7	Urinalysis		
Alb	3.8 g/dl	3.1–4.8	U-protein	(-)	(-)
BUN	6 mg/dl	5–27	U-glucose	(-)	(-)
Cre	0.40 mg/dl	0.30–0.90	U-occult blood	(-)	(-)

Initially, the patient had been diagnosed with SLE and had received prednisolone and mizoribine treatment. Although symptoms ameliorated immediately, CH50 remained consistently negative during a period of six months, whereas C3 and C4 were within the normal range. Antinuclear antibody and rheumatoid factor levels gradually elevated, but anti-double stranded DNA IgG antibody levels were still negative. From the above discrepancy between symptoms and laboratory data changes, we suspected complement deficiency so investigated her complement system in detail. Serum C1q levels measured at SRL Inc. (Tokyo, Japan) by nephelometry were 2.8 mg/dl (normal range: 8.8–15.3 mg/dl). Further analysis of the complement systems revealed that whole alternative complement pathway activity (ACH50) and C4 and C2 activities were within the normal range, whereas C1 activity was very low (Table 2) [10-13]. The addition of purified human C1 subcomponent enabled CH50 to be restored to a normal range only when C1q was added, but neither C1r nor C1s was effective (Table 3). For this reason, we strongly suspected a C1q deficiency.

**Table 2:** Analysis of complement system activity

**Hemolytic activity**	**NHS**	**Patient serum**	**Reference value**
CH50 (U/ml)	125	0	90–160
(NHS %)	100%	0%	
ACH50 (U/ml)	18.5	18.9	
(NHS %)	100%	102%	70–130%
C1 activity (U/ml)	1450	100	
(NHS %)	100%	7%	70–130%
C2 activity (U/ml)	400	360	
(NHS %)	100%	90%	70–130%
C4 activity (U/ml)	2000	1600	
(NHS %)	100%	80%	70–130%

*NHS *normal human serum, *ACH50 *alternative complement pathway activity.

**Table 3:** Addition of purified human C1 subcomponent

**Hemolytic activity**	**NHS**
CH50 (U/ml)	125
(NHS %)	100%
Patient serum + C1q (U/ml)	140
(NHS %)	112%
Patient serum + C1r (U/ml)	0
(NHS %)	0%
Patient serum + C1s (U/ml)	0
(NHS %)	0%
Patient serum + activated C1s (U/ml)	0
(NHS %)	0%

*NHS *normal human serum.

**Figure 2:**
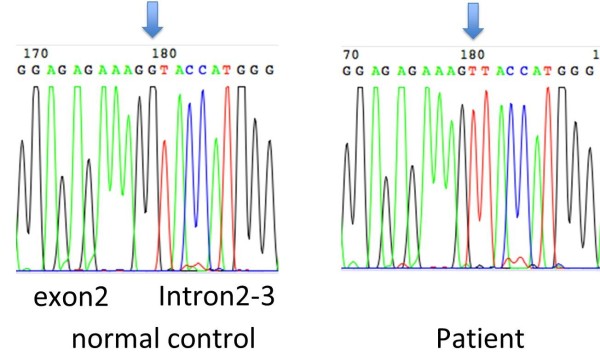
**Sequence analysis of the *C1qB *gene.** A homozygous mutation was identified at the consensus splicing donor site in intron 2–3 caused by a G-to-T transversion (c.187 + 1G > T).

**Figure 3:**
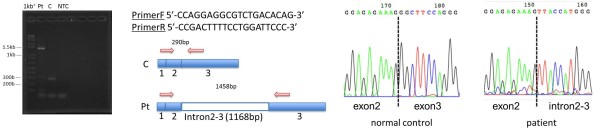
**Reverse transcriptase-polymerase chain reaction analysis of *C1qB *mRNA.** A single band observed at 1458 bp was caused by a splicing error of intron 2–3. 1 kb+, 1,000 bp DNA-ladder marker; Pt., patient; C., normal control; NTC, no template control.

**Figure 4:**
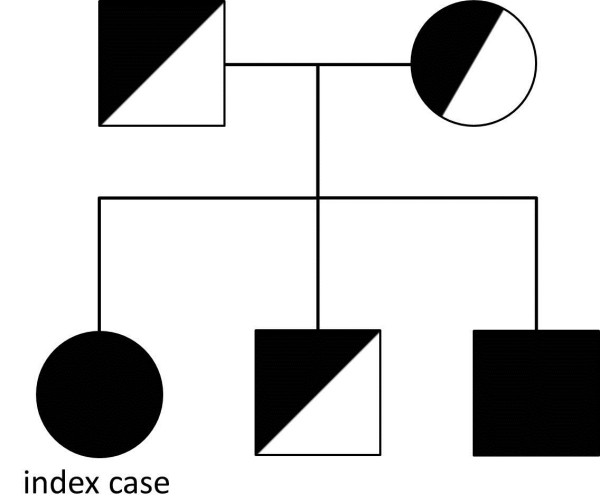
Pedigree showing the heterozygosity of the patient’s parents and older brother, and the homozygosity of her younger brother, for c.187 + 1G > T.

Genomic DNA was extracted from EDTA-blood cells using standard procedures. PCR primers were designed to amplify all exons and exon-intron boundaries of *C1qA*, *C1qB* and *C1qC* genes. Table 4 shows the primer sets and sequencing performed with the BigDye terminator v3.1 Cycle Sequencing Kit. Sequence analysis revealed a novel homozygous splice site mutation in *C1qB*, c.187 + 1G > T (Figure 2), but no other mutations. Reverse transcriptase-polymerase chain reaction amplification of *C1qB* demonstrated the presence of an abnormal single band on gel electrophoresis caused by a splicing error of intron 2/intron 3 (Figure 3). Combined with the complement assay, this DNA sequencing result enabled the molecular diagnosis of C1q deficiency to be confirmed. Mutation analysis and CH50 measurements of family members demonstrated that the patient’s parents and one sibling were heterozygous for c.187 + 1G > T with normal CH50 levels. The second sibling (0 years old) with undetectable CH50 levels was also homozygous for the mutation (Figure 4).

**Table 4:** List of primers used in this work

**Primer name**	**Gene**	**Exon**	**F/R**	**Primer sequence (5’-3’)**	**Tm (°C)**	**Annealing Tm (°C)**
C1QA-02-F	C1qA	02	F	TTGTGTGCATGGGACTCAAG	56	60
C1QA-02-R	C1qA	02	R	GGCCAAGTCAGGCCAAG	58	
C1QA-03a-F	C1qA	03a	F	TCCCTGAGGACCAGTAGGC	60	60
C1QA-03a-R	C1qA	03a	R	GGACAGGCAGATTTCCCAC	58	
C1QA-03b-F	C1qA	03b	F	TCATCTTCGACACGGTCATC	56	60
C1QA-03b-R	C1qA	03b	R	ATTTTACAGGCGGAGCATGG	56	
C1QB-02-F	C1qB	02	F	GGATGCAGATGGAGGGATAG	58	60
C1QB-02-R	C1qB	02	R	AGGCAACTGTGACTTGGGAG	58	
C1QB-03a-F	C1qB	03a	F	GCAGGCCTCCTTCTTTTGG	58	60
C1QB-03a-R	C1qB	03a	R	TCACGCACAGGTTCCCTC	58	
C1QB-03b-F	C1qB	03b	F	CAGACCATCCGCTTCGAC	58	60
C1QB-03b-R	C1qB	03b	R	GGGGTAGAGTGAGCGTTGC	60	
C1QC-02-F	C1qC	02	F	ATCCATGGTGAGGCTCCTG	58	60
C1QC-02-R	C1qC	02	R	CCCAGACAGACACTCTGATCC	60	
C1QC-03a-F	C1qC	03a	F	GTTCCCTGGAAGACACCCTC	60	60
C1QC-03a-R	C1qC	03a	R	TATGCGACGCGTGGTAGAC	58	
C1QC-03b-F	C1qC	03b	F	AGCCTGATCAGATTCAACGC	56	60
C1QC-03b-R	C1qC	03b	R	TGGCCAGTAAGGTGGGTCC	60	

*F *forward, *R *reverse, *Tm *temperature.

After about one year from the first visit, the patient experienced a recurrent fever every few days with no sign of infection. Despite increasing the doses of prednisolone (15 mg/day) and mizoribine (300 mg/every other day), fever and erythematosus rash were aggravated and the ESR elevated to 68 mm/60 min, which is the index that most reflects her condition. We carried out intravenous methylprednisolone pulse therapy, but the effect was only temporary. Accordingly, we initiated fresh frozen plasma (FFP) transfusion for supplementation of C1q. Immediately after transfusion of FFP (10 ml/kg), her CH50 levels recovered to within the normal range but became undetectable 24 h later (Table 5). Nevertheless, she remained afebrile and her rash improved slowly, while ESR declined to 37 mm/60 min following once weekly administration of FFP (10 ml/kg). We are currently attempting to slowly reduce the prednisolone dosage and the frequency of FFP.

**Table 5:** Measurement of CH50 following transfusion of FFP in the patient

**Time (hr)**	**0**	**3 (end of transfusion)**	**9**	**24**
CH50 (U/ml)	<12.0	25.5	24.6	<12.0

## Conclusions

We report a girl diagnosed with SLE because of C1q deficiency caused by a novel homozygous splice site mutation in *C1qB*. C1q deficiency is a rare autosomal recessively inherited disease, with only 41 patients from 23 families reported in 1998 [2]. More recently, C1q deficiency has been confirmed in 64 cases within 38 families, 88% of which present with SLE or SLE-like disease [7].

There are three hypotheses regarding the relationship between C1q deficiency and SLE or SLE-like disease. The first is that C1q deficiency causes autoimmunity by impairing the clearance of apoptotic cells [2], while in the second the absence of C1q affects the negative selection of autoreactive B cells [14]. The third is that the lack of C1q leads to increased interferon-α production and the defective regulation of dendritic cells [15]. It has also been reported that C1q activates canonical Wnt signaling, which regulates T cell development and dendritic cell maturation [16-18]. The suppression of Wnt signaling in association with C1q deficiency may result in the inadequate activation of lymphocytes.

The majority of C1q deficiency patients are European or Middle Eastern, with only four cases reported to date in Japan, none of which were confirmed by genetic analysis [7,19-22]. Al-Mayouf et al. reported that C1q deficiency patients tend to develop SLE in less than five years [4]. Our patient also developed SLE at the age of four, with persistent hypocomplementemia and normal C3 and C4 levels. We believe that the possibility of C1q deficiency should therefore always be considered in such cases. To our knowledge, 14 different C1q deficiency-causing mutations have been identified to date, most in European and Middle Eastern patients, with one in an African-American ancestry [7-9]. All are missense or nonsense mutations, so the present mutation is the first report of a splicing error associated with the disorder.

Previously reported complications of C1q deficiency include glomerulonephritis in 30%, central nervous system involvement in 19%, and bacterial infections in 41% of patients [7]. Moreover, symptoms present with varying degrees of severity, even in the same family [23]. It is possible that environmental factors such as exposure to ultraviolet radiation or a history of infection may affect the epigenetic regulation of developing SLE-like syndrome. In our case, the patient may develop complications later in life, and her homozygous sibling may also develop SLE. It is therefore important to carefully follow up our patients to prevent the onset of glomerulonephritis and central nervous system involvements.

At present, no specific therapy is available for C1q deficiency, so steroids and immunosuppressive agents such as hydroxychloroquine (not currently available in Japan) are used as treatments. Bone marrow transplantation is a potential cure, but it has not yet been performed in humans [24,25]. Some reports show that the infusion of FFP restores C1q levels, temporarily complements hemolytic activity, and suppresses SLE symptoms for a long period [8,24]. It is therefore a valid therapy for C1q deficiency patients. However, the long-term administration of FFP increases the risk of infection and the possibility of side effects caused by the development of anti-C1q antibodies [26]. In the light of such risks, alternative treatment strategies should be considered for this intractable disease.

## Consent

Written informed consent was obtained from the patient for publication of this Case Report and any accompanying images. A copy of the written consent is available for review from the Editor-in-Chief of this journal.

## Abbreviations

SLE: Systemic lupus erythematosus; ESR: Erythrocyte sedimentation rate; CRP: C-reactive protein; CH50: Total complement activity; CT: Computed tomography; FFP: Fresh frozen plasma.

## Competing interests

The authors have nothing to disclose.

## Authors’ contributions

YH drafted the manuscript, and participated in treatment. JS, KA, and TK carried out the clinical treatment and helped draft the manuscript. MH, EK, and HK analyzed the complement systems and interpreted the data. HT, MI, TH, and OO sequenced the *C1q* genes and performed reverse transcriptase-polymerase chain reaction analysis of *C1qB* mRNA. All authors read and approved the final manuscript.
